# Novel mutations in *NEB *cause abnormal nebulin expression and markedly impaired muscle force generation in severe nemaline myopathy

**DOI:** 10.1186/2044-5040-1-23

**Published:** 2011-06-20

**Authors:** Michael W Lawlor, Coen A Ottenheijm, Vilma-Lotta Lehtokari, Kiyomi Cho, Katarina Pelin, Carina Wallgren-Pettersson, Henk Granzier, Alan H Beggs

**Affiliations:** 1 Division of Genetics and Program in Genomics, The Manton Center for Orphan Disease Research, Children's Hospital Boston, Harvard Medical School, 300 Longwood Avenue, CLSB 15026, Boston, MA 02115, USA; 2 Department of Physiology, University of Arizona, 1501 N. Campbell, Rm. 4104, Tucson, AZ, 85724, USA; 3 Laboratory for Physiology, Institute for Cardiovascular Research, VU University Medical Center, Van der Boechorststraat 7, Amsterdam 1081 BT, The Netherlands; 4 The Folkhälsan Institute of Genetics and Department of Medical Genetics, Haartman Institute, P.O. Box 63 (Haartmaninkatu 8), FI-00014, University of Helsinki, Helsinki, Finland; 5 Division of Genetics, Department of Biosciences, P.O. Box 56 (Viikinkaari 9), FI-00014, University of Helsinki, Helsinki, Finland

**Keywords:** congenital myopathy, nemaline myopathy, nemaline rod (body), thin filament, nebulin

## Abstract

**Background:**

Nemaline myopathy (NM) is a congenital muscle disease associated with weakness and the presence of nemaline bodies (rods) in muscle fibers. Mutations in seven genes have been associated with NM, but the most commonly mutated gene is nebulin (*NEB*), which is thought to account for roughly 50% of cases.

**Results:**

We describe two siblings with severe NM, arthrogryposis and neonatal death caused by two novel *NEB *mutations: a point mutation in intron 13 and a frameshift mutation in exon 81. Levels of detectable nebulin protein were significantly lower than those in normal control muscle biopsies or those from patients with less severe NM due to deletion of *NEB *exon 55. Mechanical studies of skinned myofibers revealed marked impairment of force development, with an increase in tension cost.

**Conclusions:**

Our findings demonstrate that the mechanical phenotype of severe NM is the consequence of mutations that severely reduce nebulin protein levels and suggest that the level of nebulin expression may correlate with the severity of disease.

## Background

With an estimated incidence of 1 in 50,000 live births, nemaline myopathy (NM) is the most common of the congenital myopathies, accounting for roughly one-half of the cases of these conditions [1]. Clinically, NM is heterogeneous, producing symptoms ranging from profound perinatal weakness and hypotonia to mild, nonprogressive weakness with onset in adolescence or adulthood. A diagnosis of NM requires symptoms of skeletal muscle weakness and the presence of nemaline rods in muscle fibers, in the absence of findings diagnostic of other unrelated conditions [1]. To date, mutations of seven genes have been implicated in NM, including tropomyosin 3 (*TPM3*) [2], skeletal α-actin (*ACTA1*) [3], nebulin (*NEB*) [4], tropomyosin 2 (*TPM2*) [5], troponin T (*TNNT1*) [6], cofilin 2 (*CFL2*) [7] and *KBTBD13 *[8]. With the exception of *KBTBD13*, whose function is unknown, these genes share the unifying feature that they all encode proteins of the sarcomeric thin filament, suggesting that weakness and rod formation in NM is related to improper thin-filament structure and function [9].

The skeletal muscle-specific *NEB *gene is large, with a total of 183 exons spanning 249 kb of genomic sequence and a theoretical full-length transcript of 26 kb, and is predicted to encode an approximately 800-kDa protein [10]. Great diversity in nebulin size, and possibly function, is generated through alternative splicing of at least 41 *NEB *exons, leading to production of at least hundreds of distinct isoforms [11,12]. A single nebulin molecule spans the thin filament with its C terminus anchored at the Z-disk and its N-terminal region directed toward the pointed end of the thin filament. Recent studies have suggested that nebulin may play a role in the regulation of contraction in addition to its role in determining thin-filament length [13]. Control of thin-filament length is essential for proper muscle contraction, since the degree of overlap between thick and thin filaments determines the amount of force that a muscle can produce [4,14-17]. Nebulin enhances force generation by altering cross-bridge cycling kinetics to increase the number of force-generating cross-bridges [18,19]. Studies of two nebulin-knockout mouse models, both of which exhibit severe, early, lethal phenotypes, have shown that nebulin absence is associated with shorter thin filaments [18,20,21] and altered cross-bridge cycling [22].

Mutations of the skeletal muscle-specific *NEB *gene are the most common cause of autosomal recessive NM [23]. Although often associated with the nonprogressive or slowly progressive "typical" form of congenital NM [24], *NEB *mutations have also been reported in patients with "intermediate" and "severe" forms of NM, characterized by lack of ambulation or even death in infancy [24]. To date, 64 different mutations in *NEB *have been reported in NM probands [4,10,14]. Largely because that portion of the gene was studied first, many of the known mutations reside in the 3' end of the gene and may affect interactions between nebulin and other proteins at the Z-disc. Some cases exhibit loss of immunoreactivity of some proximal epitopes, with retention of distal epitopes, suggesting that complex patterns of alternative or abnormal splicing allow production of internally deleted, but stable and partially functional, proteins [4,17]. These studies focused on the presence of immunohistochemical staining in patient muscle biopsies, however, and did not assess the quantity of nebulin present. A relatively common in-frame deletion of *NEB *exon 55, identified in Ashkenazi Jewish NM patients with variable forms of NM, has been studied extensively at the genetic and physiological levels and has been shown to result in moderately reduced levels of nebulin [10,15,17]. In this report, we describe clinical, histological, genetic, protein expression and muscle fiber contractility studies in a sibling pair with multiple congenital contractures and neonatal death due to a particularly severe form of NM resulting from two compound heterozygous mutations in *NEB*.

## Results

A North American family (family "16") with two affected siblings with severe NM was referred for research studies to determine the genetic basis for their condition.

### Parental history

At the time of the birth of patient 16-2, the mother was 25-year-old G7, P2-2-2-4 (seven pregnancies, two term births, two preterm births, two abortions and four living children). There was no family history of neuromuscular disease in either parent, and neither parent had signs or symptoms of muscle disease.

### Patient 16-2

This baby boy was born after a pregnancy complicated after 31 weeks by polyhydramnios, and fetal movements were weak and infrequent. Birth occurred at 37 weeks gestational age, and the boy required intubation in the delivery room. He was 47 cm in length, weighed 2,500 g, and had a head circumference of 37.5 cm. The patient had facial weakness; contractures of the hips, knees, ankles, elbows and wrists; and other abnormalities, including a broad, prominent forehead; downward-slanting palpebral fissures; micrognathia; a bulbous nose; a cleft palate; ears that were low-set and posteriorly rotated; cryptorchism; and a small phallus. His neurological findings were otherwise normal. He required tube feeding. Echocardiography revealed a large patent ductus arteriosus with pulmonary hypertension. Electromyography performed at one week of age was inconclusive. A biopsy of the right rectus femoris muscle obtained at eight days of life revealed myopathic muscle with numerous nemaline bodies and/or rods, which are diagnostic for NM (see below). He was ventilator-dependent until 28 days of life, when ventilator care was withdrawn and he was taken home. He died a few hours thereafter.

### Patient 16-4

This baby boy was born two years later to the same parents at 31 weeks gestational age by spontaneous vaginal delivery, with Apgar scores of 1, 1 and 2 at one, five and ten minutes, respectively. The pregnancy was complicated by oligohydramnios and preterm precipitous onset of labor. *In utero *monitoring demonstrated poor fetal movement and contractures of the upper and lower extremities. At birth, contractures were present at the elbows, wrists, fingers, hips, knees and feet, with the first, second and fifth digits overlapping the third and fourth digits of the hands. The infant had significant respiratory distress at delivery, with no respiratory effort and poor color despite administration of 100% oxygen. Prior to delivery, the parents had requested supportive measures only, and active treatment was discontinued because of the patient's clinical findings and significant respiratory distress during his first day of life. The patient died shortly thereafter.

### Pathological studies

#### Patient 16-2

**Figure 1:**
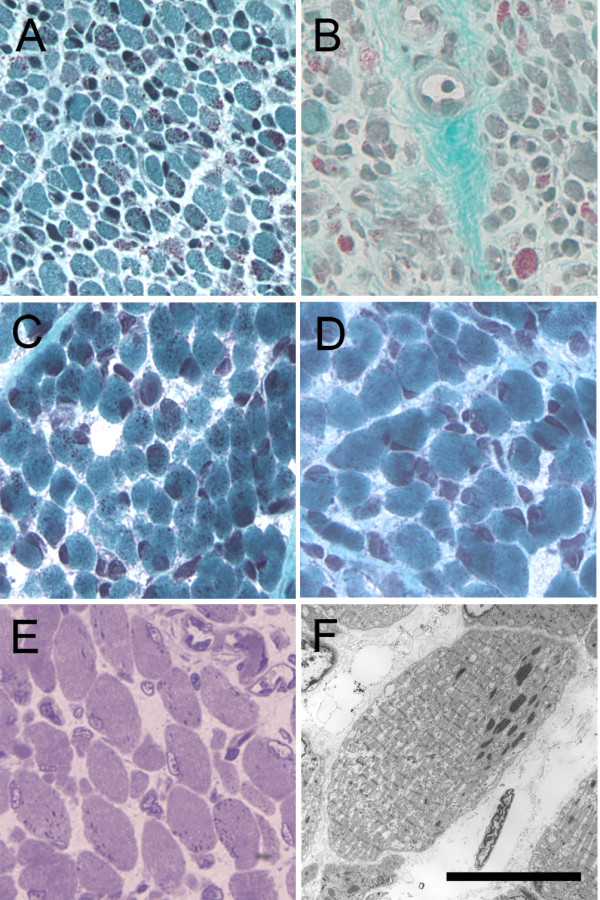
**Histological findings in two brothers with severe NM**. Gomori trichrome staining **(A) **through **(D) **of frozen muscle tissue reveals punctate red inclusions (nemaline rods) within the cytoplasm of skeletal muscle fibers. These structures are readily identifiable in a biopsy specimen from the rectus femoris muscle of patient 16-2 **(A)**, an autopsy specimen of the psoas muscle from patient 16-2 **(B) **and autopsy specimens from the diaphragm **(C) **and abdominal wall **(D) **muscles of patient 16-4. Toluidine blue staining of Epon-embedded quadriceps muscle from patient 16-2 **(E) **reveals a diffuse distribution of nemaline rods and marked variation in myofiber size. Note the variation in nemaline rod burden, fiber size variation and fibrosis between individual muscles within the same patient. Ultrastructural examination of this tissue **(F) **confirms the identity of the dense cytoplasmic inclusions as nemaline rods. Scale bars = 200 μm for **(A) **through **(E) **and 40 μm for **(F)**. NM, nemaline myopathy.

A right rectus femoris muscle biopsy taken at eight days of life revealed skeletal muscle with small, round fibers and excessive variation in fiber size (Figure 1A). Numerous cells of all sizes and fiber types contained granular red material in a diffuse cytoplasmic and subsarcolemmal distribution, consistent with the presence of nemaline rods. No nuclear rods were seen. There was a mild focal increase in perimysial fibrosis. The nicotinamide adenine dinucleotide (NADH) and succinic dehydrogenase (SDH) stains revealed type 1 fiber predominance with appropriately sized type 2 fibers. No inflammation, excessive central nucleation, corelike structures or myonecrosis was seen.

The causes of death reported on the basis of autopsy findings were severe congenital NM and patchy acute bronchopneumonia of the left lung. The lungs appeared mildly edematous but without discrete lesions on the cut surface. The heart and all other organs were reportedly unremarkable. The histological findings in cardiac muscle were normal at both light and ultrastructural levels.

Frozen muscle from the psoas, quadriceps, diaphragm and cardiac muscles collected at the time of autopsy were available for histological review. Gomori trichrome staining of the psoas muscle (Figure 1B) revealed skeletal muscle with small, round fibers and excessive variation in fiber size. The findings in this specimen were similar to those seen in the patient's earlier biopsy, except that the degree of perimysial and endomysial fibrosis was more marked here and a greater proportion of the fibers (approximately 80% to 90%) contained a diffuse distribution of nemaline rods. No nuclear rods were seen. The quadriceps muscle had moderate to severe variation in fiber size, mild perimysial fibrosis and a diffuse and subsarcolemmal distribution of nemaline rods in some fibers (Figure 1E). Nemaline rods were found in fewer fibers of the quadriceps muscle compared to the psoas muscle. Ultrastructural examination of the quadriceps muscle further confirmed the presence of nemaline rods in muscle fibers (Figure 1F). Focal Z-band streaming was also present. The mitochondria were appropriate with respect to their size, shape, complexity and distribution. The diaphragm displayed marked variation in fiber size with scattered round small fibers and a mild, focal increase in perimysial fibrosis. Most muscle fibers in the diaphragm were much larger than the fibers seen in any of the other specimens from this patient, including the prior muscle biopsy. Scattered fibers contained subsarcolemmal aggregates of granular red material, suggestive of nemaline rods. It should be noted, however, that these aggregates were much smaller and less numerous than those seen in the other skeletal muscle specimens. No inflammation, corelike structures or myonecrosis was seen in any of the specimens.

#### Patient 16-4

At autopsy, pulmonary findings included bilobed right lung, markedly hypoplastic lungs bilaterally, and immature, minimally expanded lung parenchyma. Histologically, minimally expanded alveoli contained scattered squamous cells and possible early hyaline membranes. The right and left pulmonary veins were significantly smaller than expected (1 or 2 mm), but the relationship and size of the vasculature were otherwise normal. Sections taken from the thymus, trachea, esophagus, adrenal gland, spleen, kidney, pancreas, bone, bone marrow and brain were unremarkable when visualized by light microscopy. The heart was structurally normal for gestational age, with a patent ductus arteriosus and foramen ovale.

Evaluation of skeletal muscle from the diaphragm (Figure 1C) revealed small, round myofibers containing large, peripherally placed nuclei consistent with neonatal muscle. Gomori trichrome staining revealed numerous nemaline rods in most myofibers, with a diffuse distribution of rods within the myofibers. NADH and ATPase staining allowed only poor differentiation between oxidative and glycolytic fibers, but nemaline rods were present in both fiber types. Skeletal muscle from the abdominal wall (Figure 1D) showed increased variation in fiber size, with numerous small, round fibers and a greater degree of fiber size variation than was seen in the diaphragm. The myofibers had large, peripherally placed nuclei with coarse granular basophilic staining in many fibers. Gomori trichrome staining revealed nemaline rods within many fibers, which were arranged in a predominantly diffuse distribution within myocytes. The rods were readily found in both large and small fiber populations. NADH and ATPase staining revealed that the small fibers were of both oxidative and glycolytic fiber types, and nemaline rods were located within both fiber types.

### Mutation analysis

**Figure 2:**
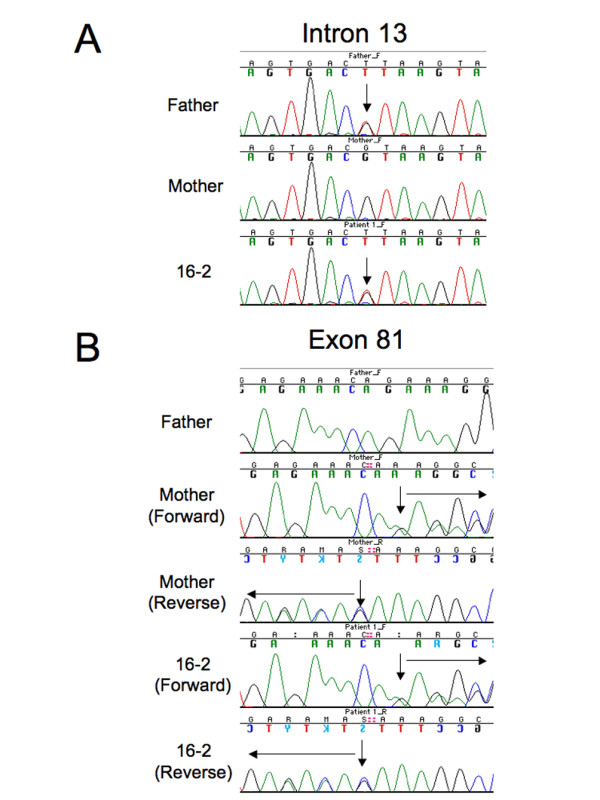
**Severe NM in two brothers caused by compound heterozygous mutations for an exon 13 splice site and an exon 81 frameshift**. DNA sequence analysis of genomic PCR products illustrates the two mutations found in these patients. **(A) **A splice site mutation in the 5' splice site of intron 13 (GT > TT)(g.32596G > T) was found in both reported patients and their father, but was not seen in their mother. **(B) **A deletion of two nucleotides was found in exon 81 (g.129384_129385del) of both patients and their mother, but was not present in their father. The results of PCR assays run in forward and reverse are shown to confirm the presence of a frameshift mutation. The sequencing results for patient 16-4 were identical to those shown for patient 16-2. Arrows indicate mutation sites.

Genetic studies of the *ACTA1, TPM2, TPM3 *and *CFL2 *genes, as well as the recurrent *NEB *exon 55 deletion, were all negative in one or the other of the two affected patients. Genomic PCR and denaturing high-performance liquid chromatography (dHPLC) analysis of 159 *NEB *gene (GenBank:NG_009382.1) exons revealed two distinct mutations, one each in the patients' father and mother. Both affected boys were compound heterozygotes for these two autosomal recessive mutations. The mutation inherited from the father was a splice site mutation in the 5' splice site of intron 13 (GT > TT) (g.32596G > T, c.1152 + 1G > T) (Figure 2A), and the mutation inherited from the mother was a deletion AG leading to a frameshift in exon 81 (g.129384_129385del, c.11318_11319del, p.Lys3774Argfs*10) (Figure 2B). Neither of these mutations was identified in 236 control chromosomes analyzed by sequencing. Exons 13 and 81 are both constitutively expressed, so patients with this combination of mutations would not be expected to produce normal nebulin.

### Nebulin expression

**Figure 3:**
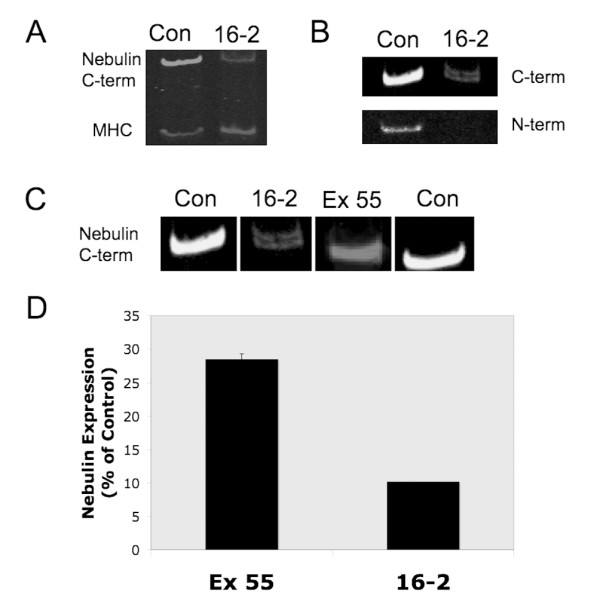
**Compound heterozygosity for the exon 13 and exon 81 mutations is associated with dramatic reduction of nebulin protein levels in NM muscle**. **(A) **Nebulin expression in comparison to myosin heavy chain (MHC) expression is shown using extracted protein from a control patient (Con) or from patient 16-2 (16-2). **(B) **Nebulin was detected using antibodies directed against either the N-terminal (N-term) or the C-terminal (C-term) regions of nebulin. Only the C-terminal antibody reacted with nebulin in the NM sample and reveals a barely detectable doublet. **(C) **Comparison of nebulin expression in control patients (Con), patient 16-2 (16-2) and a patient with nemaline myopathy caused by a mutation in exon 55 (Ex 55). **(D) **Quantification of nebulin expression (normalized to MHC expression and expressed as a percentage of the values seen in controls) in four patients with nemaline myopathy caused by mutations in exon 55 in comparison to the expression measured in patient 16-2. Values shown are means ± standard error of the mean (SEM).

Western blot analysis performed using protein extracted from quadriceps muscle from patient 16-2 revealed significantly lower nebulin content in comparison to control patients and patients with exon 55 mutations who had been evaluated in a prior report [17]. Protein from patient 16-4 could not be evaluated. The antibody against nebulin's N terminus showed no detectable labeling, whereas the antibody against nebulin's C terminus detected a nebulin isoform at the appropriate molecular weight (approximately 773 kDa), similar to what was seen in control samples (Figure 3A). However, when normalized to myosin heavy chain (MHC), labeling of nebulin's C terminus was approximately 90% less intense in the NM patient compared with control (Figure 3D). Also, the antibody against nebulin's C terminus detected a doublet (Figure 3C), which might be a reflection of the different mutations on each of the alleles. In contrast, four patients with NM due to homozygous deletion of exon 55 of *NEB *showed labeling of nebulin's C terminus approximately 72% reduced in comparison to control [17]. Prior work using these samples [10,17] described a less severe clinical course and less severe abnormalities seen on contractile studies using myofibers from these patients.

### Muscle mechanics

**Figure 4:**
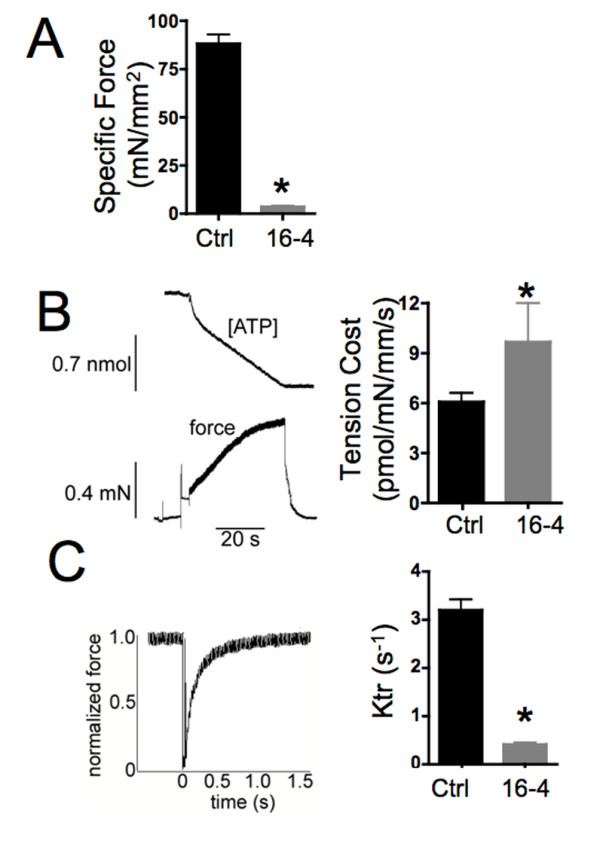
**Weakness in severe NM with greatly reduced nebulin levels is associated with reduced force generation with increased tension cost and slower force redevelopment**. Skinned myofibers from control patients (Ctrl) and patient 16-4 (16-4) reveal **(A) **a large reduction in maximal tension measured at -log[Ca2+] (pCa) 4.5, **(B) **an increase in tension cost and **(C) **a large reduction in the rate constant of force development (*k*_tr_). Control myofibers were taken from three normal quadriceps biopsies, and analysis was limited to type 2 fibers, since the patient 16-4 biopsy expressed mainly type 2 myosin heavy chains. Values are the means ± SEM of measurements from five myofibers. *P < 0.05.

Recent studies using tissue from nebulin-knockout mice [18-22] and humans with NM due to deletions of *NEB *exon 55 [17,22] have demonstrated an impaired capacity to generate force because of shorter thin filaments and altered cross-bridge cycling. Here we used skinned fiber preparations to determine the contractile properties of myofibers from patient 16-4, whose clinical presentation and quantity of nebulin represent an intermediate phenotype between the knockout mice and patients with the exon 55 deletion. Fibers isolated from a diaphragm specimen had severely reduced maximal Ca^2+^-activated active tension (4 ± 0.5 mN/mm^2 ^compared with 88 ± 5 mN/mm^2 ^in control) (Figure 4A), which is similar to the recent report of contractile function in fibers of NM patients with *NEB *exon 55 deletions [17]. Because of the use of autopsy tissue in these studies, we cannot rule out that postmortem protein degradation contributed to the decrease in contractile performance, but the decrease was consistent among fibers and similar in scale to findings in our previous studies of surgical specimens. The rate of force redevelopment (*k*_tr_) was dramatically decreased from 3.2 ± 0.2 s^-1 ^in control myofibers to 0.4 ± 0.04 s^-1^, even more so than in the previously described patients [17] (Figure 4C). There was also a significant increase in tension cost (adenosine triphosphate (ATP) consumption rate normalized to force) in fibers isolated from our patients (9.7 ± 2.3 pmol/mN/mm/second) in comparison to controls (6.1 ± 0.6 pmol/mN/mm/second) (Figure 4B). These findings are consistent with severely impaired force generation that can be partly explained by altered cross-bridge cycling kinetics (slower cross-bridge attachment and faster detachment), resulting in a lower fraction of bound force-generating cross-bridges [17,22].

## Discussion

While a number of *NEB *mutations have been described in patients with NM, the differences in molecular pathogenetic pathways leading to variable disease severity are currently unclear. Here we have described two siblings with compound heterozygote mutations in intron 13 and exon 81 of *NEB*, resulting in severe congenital myopathy with profound muscle weakness, arthrogryposis and neonatal death. The splice site mutation in intron 13 is expected to cause exon 13 skipping, which would result in a protein lacking 39 amino acids in the N terminus disrupting simple repeat M8 and super repeat 1. The frameshift mutation in exon 81 is predicted to cause premature truncation of the protein at super repeat 14. These mutations were associated with a marked decrease in the quantity of nebulin detectable by Western blot analysis, which was found to be at even lower levels than those seen in patients with severe NM due to exon 55 mutations in *NEB *[17]. Pathologically, the patient muscles showed a variable degree of myopathic changes and nemaline body (rod) burden. Contractility testing of skinned myofibers from the diaphragm revealed marked deficits in contractile performance.

Mutations in *NEB *are the most common cause of NM [4]. While the clinical findings in *NEB*-associated NM are variable, the most frequent presentation of these patients is the so-called "typical" form of NM. Patients with typical NM have congenital onset of weakness followed by delayed attainment of gross motor milestones and a slowly progressive or nonprogressive course. Although the severe form of NM, which is associated with a lack of spontaneous movements or respiration at birth, sometimes with multiple congenital contractures or fractures [25], is more frequently caused by mutations in *ACTA1 *[24], severe NM has been reported in 13 families as a consequence of *NEB *mutation [14,24]. In the first report of severe NM caused by *NEB *mutation, three of five families had mutations located in exon 184 [24], suggesting that this might represent a hotspot for mutations causing severe disease. However, none of the subsequently reported families had mutations of this exon [14], although, notably, one of these families had a splice site mutation in intron 81 predicted to lead to a defect similar to the exon 81 frameshift found in our family 16.

The particularly severe phenotype of both affected children in our study is reflected by the presence of multiple contractures at birth, or arthrogryposis multiplex congenita (AMC). Rather than a specific pathological diagnosis, AMC is a description of a clinical phenotype that occurs in 1 in 3,000 live births and is a characteristic of more than 300 different disorders [26]. In cases of AMC that are associated with neuromuscular disease, contractures are present at birth as a result of fetal muscle weakness causing insufficient movements. AMC has been reported in some cases of severe NM due to mutations in *NEB *[25], but this is an uncommon and particularly severe presentation of this disease. Our data suggest that low levels of nebulin may be associated with such a severe NM presentation and AMC and also that quantitation of the nebulin content in skeletal muscle might be useful in the workup of patients with AMC.

A recent report described variably severe NM in patients with mutations in exon 55 of *NEB*, which encodes an N-terminal portion of nebulin [10]. Mechanical and structural analyses of muscle from these patients led to the discovery that the exon 55 deletions produced decreased contractile force because of shorter and nonuniform thin-filament lengths, despite a lack of significant changes in the molecular weight of nebulin [17]. Contractile studies of *Neb*-knockout mice [27], the patients with exon 55 deletions [17,22] and our more severely affected patients described herein all showed large reductions in maximal tension and *k*_tr _and increases in tension cost. Whether the apparently greater diminution of *k*_tr _in muscle from patient 16-4 is really associated with these boys' unusually severe clinical presentations will require the identification and analysis of additional similar cases.

Our Western blot studies using antibodies against the N- and C-terminal portions of nebulin revealed significant reduction of nebulin content with greater immunoreactivity detected using antibodies against the C-terminal portion of nebulin rather than the N-terminal portion. Studies of patients with deletion of exon 55 have also detected reduced quantities of nebulin of appropriate molecular size and weaker immunoreactivity to antibodies directed against the N terminus [17]. These findings confirm earlier results in patients with other nebulin mutations and suggest that mutations affecting the N-terminal regions of nebulin can impair recognition of nebulin using antibodies against the N terminus while leaving C-terminal immunoreactivity intact [4,28,29]. Notably, the overall quantity of nebulin in muscle from patient 16-2 (estimated to be 10% of normal) was markedly lower than the approximately 28% levels seen in four patients with exon 55 deletion, suggesting that the greater severity of symptoms in our patients might be related to the degree of nebulin deficiency. In contrast, a recent report described clinical and mechanical findings in an NM patient with > 70% normal levels of nebulin due to compound heterozygosity for two splicing mutations predicted to induce skipping of *NEB *exons 3 and 22 [30]. In that study, the patient was a 46-year-old man with the "typical" form of NM, with only mild (Medical Research Council (MRC) grade 4) weakness. Remarkably, and in contrast to our findings in patients with a greater degree of nebulin deficiency, muscle from this patient exhibited a normal force-sarcomere length relationship and normal calcium sensitivity of force production. A more complete correlation between nebulin expression and clinical severity is necessary, but, taken together, these data suggest that levels of nebulin expression detected by Western blot analysis may be indicative of the underlying molecular mechanisms of weakness and thus may be useful in predicting the prognosis of patients with NM due to mutations in *NEB*.

## Conclusions

Our studies provide further evidence that *NEB *mutations can cause marked impairment of contractile performance that causes severe myopathic disease. The compound heterozygous mutations described in this report represent another scenario in which *NEB *mutations can cause severe NM that is associated with an even shorter life expectancy than that of the most severely affected patients with exon 55 mutations. Single-fiber contractile studies from patient 16-2 in this study revealed marked decreases in contractile force compared with control fibers, and these decreases exceeded the contractile force deficits reported in studies using myofibers from NM patients with mutations in exon 55 of *NEB *[22]. Overall, the cases described in this study represent the severe end of the clinical spectrum of NM, exceeding the clinical and physiological deficits observed in patients with deletion of exon 55 of *NEB*. Additionally, our results imply that low levels of nebulin protein detected by Western blot analysis may correlate with a poor prognosis for patients with NM due to *NEB *mutations, which would be diagnostically useful if this finding holds true in studies of larger numbers of patients.

## Methods

### Pathological evaluation

Muscle biopsy and autopsy tissues were obtained and prepared using standard histological protocols [31]. The rectus femoris, psoas, quadriceps, diaphragm and cardiac muscles were obtained from patient 16-2, and the diaphragm and abdominal wall muscles were obtained from patient 16-4. Briefly, fresh muscle was frozen in isopentane and stored at -80°C until sectioning. Eight-micrometer cryostat sections were stained with hematoxylin and eosin, Gomori trichrome, ATPase (at pH 4.3 and pH 9.2) or NADH. Photomicrographs were obtained by using a Nikon Eclipse 50i microscope (Melville, NY, USA) equipped with a SPOT Insight 4 Meg FW Color Mosaic camera and SPOT 4.5.9.1 software from Diagnostic Instruments (Sterling Heights, MI, USA). For electron microscopy, samples were fixed and processed according to standard histological techniques, and ultrastructural examination was performed on lead-stained, 95-nm sections at the time of autopsy and repeated during the preparation of this article.

### Mutation analysis

Mutation analysis of the *NEB *gene (GenBank:NG_009382-1) was performed by dHPLC and sequencing as previously described [14]. The dHPLC analyses were carried out using the automated Transgenomic WAVE Nucleic Acid Fragment Analysis System (Transgenomic, Omaha, NE, USA) with associated Navigator software. Primer data are available upon request (from VLL). All 159 of the 183 nebulin exons that could be analyzed by dHPLC were amplified using 148 primer pairs. PCR reactions were performed in 96-well plates suitable for dHPLC equipment. Each 35-μL reaction mixture contained 60 to 90 ng of genomic DNA (3 μL), 10× PCR buffer supplied with the AmpliTaq Gold PCR Master Mix (Applied Biosystems, Carlsbad, CA, USA) containing 15 mmol MgCl2, 5 nmol each of deoxyribonucleotide triphosphate, 20 pmol forward primer, 20 pmol reverse primer and 0.8 U AmpliTaq Gold polymerase enzyme (Applied Biosystems, Carlsbad, CA, USA). Reactions were carried out in a PTC-225 DNA Engine Tetrad Thermocycler (MJ Research, Waltham, MA, USA) starting with denaturation for 10 minutes at 95°C, followed by annealing at 55°C to 60°C depending on the amplicon and extension at 72°C. The lengths of the denaturation, annealing and extension steps varied depending on the amplicon. A final extension was performed at 72°C for 10 minutes. Amplification of the PCR products was confirmed by agarose gel electrophoresis before dHPLC analysis.

Before dHPLC analysis, PCR samples were denatured for 3 minutes at 95°C and then slowly reannealed by lowering the temperature from 95°C to 37°C over a period of 1 hour. Two to five microliters of the PCR amplicon on the 96-well plate were injected into a heated reverse-phase DNASep Column (Transgenomic, Omaha, NE, USA). The column temperature of the dHPLC was set for partially denaturing conditions. The melting profiles of the amplicons were calculated using the Navigator software, but the exact temperature was determined empirically. Conditions used for dHPLC analysis of each amplicon are available on request (from VLL).

Following dHPLC analysis, samples showing abnormal peaks were sequenced. The PCR products were purified using Exonuclease I and shrimp alkaline phosphatase (USB Corp., Cleveland, OH, USA), and the purified products were sequenced using BigDye version 3.1 sequencing chemistry and an ABI 3730 DNA Analyzer (Applied Biosystems, Carlsbad, CA, USA). Sequences were analyzed using Sequencher 4.1 software (Gene Codes Corp, Ann, Arbor, MI, USA).

### Western blot analysis

For determination of nebulin content, muscle samples (a biopsy of the quadriceps muscle from patient 16-2 and an autopsy specimen from the abdominal wall of patient 16-4) were first homogenized in buffers containing protease inhibitors (phenylmethylsulfonyl fluoride, 0.5 mmol; leupeptin, 0.04 mmol; E64, 0.01 mmol) to prevent protein degradation during the homogenization process. The homogenized muscle samples were run on 2.6% to 7% SDS-PAGE gels, and transferred onto polyvinylidene fluoride membrane using a semidry transfer unit (Bio-Rad Laboratories, Hercules, CA, USA). Blots were stained with Ponceau S to visualize total transferred protein. The blots were then probed with primary antibodies against nebulin's N terminus (rabbit polyclonal antibody, x35-x36a 1843x, provided by Dr Carol C Gregorio) and its C terminus (rabbit polyclonal antibody 6963, provided by Dr Siegfried Labeit) [21,32] or against MHC. To control for loading differences, nebulin labeling was normalized to MHC as determined from the Ponceau S-stained membrane. Secondary antibodies conjugated with fluorescent dyes with infrared excitation spectra were used for detection. One-color IR western blots were scanned (Odyssey Infrared Imaging System, LI-COR Biosciences, Lincoln, NE, USA) and the images analyzed with One-D scan EX.

### Muscle mechanics

Small strips dissected from muscle biopsies were skinned overnight at about 4°C in relaxing solution (20 mmol N,N-Bis-(2-hydroxyethyl)-2-aminoethane sulfonic acid (BES), 10 mmol ethylene glycol tetraacetic acid (EGTA), 6.56 mmol MgCl2, 5.88 mmol Na^+^-ATP, 1 mmol dithiothreitol, 46.35 mmol K^+^-propionate, 15 mmol creatine phosphate, pH 7.0, at 20°C) containing 1% (vol/vol) Triton X-100. Control samples for muscle mechanics studies were isolated from the quadriceps muscles of three living individuals between 30 and 40 years of age, and all results were comparable to previously published results for control and experimental specimens representing a variety of ages, muscle groups and postmortem or postbiopsy intervals [17,22]. To ensure that measurements were taken from representative and comparable fiber types, fibers and fiber bundles were typed and analysis was restricted to type 2 fibers (the predominant type found in the patient specimens). The skinning procedure renders the membranous structures in the muscle fibers permeable, which enables activation of the myofilaments with exogenous Ca^2+^. Preparations were washed thoroughly with relaxing solution and stored in 50% glycerol relaxing solution at -20°C for up to approximately 8 weeks. Small muscle bundles (diameter approximately 0.07 mm) were dissected from the skinned strips and mounted between a displacement generator and a force transducer element (AE 801; SensoNor, Horten, Norway) using aluminum T clips. Sarcomere length (SL) was set using a He-Ne laser diffraction system. Mechanical experiments performed on contracting muscle were carried out at a SL of about 2.5 μm for control muscle and at just over slack length for NM muscle, a length selected on the basis of our prior studies. By constructing force-SL relationships, we previously showed that at a SL of 2.5 μm, human muscle fibers from controls produced maximal force, whereas nebulin-deficient muscle fibers from NM patients produced maximal force just over slack length because of their shorter thin filaments [17]. Thus, by performing our mechanical studies on NM muscle set just over slack length, we aimed to minimize force differences due to shorter thin-filament lengths. Fiber width and diameter were measured at three points along the fiber, and the cross-sectional area was determined by assuming an elliptical cross-section. Three different bathing solutions were used during the experimental protocols: a relaxing solution, a preactivating solution with low EGTA concentration and an activating solution. The composition of these solutions was described previously [33].

### Simultaneous force-ATPase measurement

We used the system described by Stienen *et al*. to measure simultaneous force-ATPase activity [33]. To measure ATPase activity, a nearby UV light was projected through the quartz window of the bath (30 μL volume and temperature controlled at 20°C) and detected at 340 nm. The maximum activation buffer (at -log[Ca2+] (pCa) 4.5) contained 10 mmol phosphoenol pyruvate with 4 mg mL^-1 ^pyruvate kinase (500 U mg^-1^), 0.24 mg mL^-1 ^lactate dehydrogenase (870 U mg^-1^) and 20 μmol diadenosine 5'-pentaphosphate. For efficient mixing, the solution in the bath was continuously stirred by means of motor-driven vibration of a membrane positioned at the base of the bath. ATPase activity of the skinned fiber bundles was measured as follows. ATP regeneration from adenosine diphosphate (ADP) was coupled to the breakdown of phosphoenol pyruvate to pyruvate and ATP was catalyzed by pyruvate kinase, which is linked to the synthesis of lactate catalyzed by lactate dehydrogenase. The breakdown of NADH, which is proportional to the amount of ATP consumed, was measured online by UV absorbance at 340 nm. The ratio of light intensity at 340 nm (sensitive to NADH concentration) and the light intensity at 410 nm (reference signal) was obtained by means of an analog divider. After each recording, the UV absorbance signal of NADH was calibrated by multiple rapid injections of 0.25 nmol of ADP (0.025 μL of 10 mmol ADP) into the bathing solution with a stepper motor-controlled injector. The slope of the ATP concentration versus time trace during steady-state tension development of a calcium-induced contraction (Figure 4B) was determined from a linear fit, and the value was divided by the fiber volume (in cubic millimeters) to determine the fiber's ATPase rate. ATPase rates were corrected for the basal ATPase measured in relaxing solution. The ATPase rate was divided by tension (force ÷ cross sectional area (CSA) to determine the tension cost.

### k_tr _measurements

To measure *k*_tr_, we used the large slack/release approach [34] to disengage force-generating cross-bridges from the thin filaments, which were isometrically activated. Fast activation of the fiber was achieved by transferring the skinned muscle fibers from the preactivation solution containing a low concentration of EGTA (pCa 9.0) to a pCa 4.5 activating solution. Once the steady state was reached, a slack equivalent to 10% of the muscle length was rapidly induced at one end of the muscle using the motor. This was followed immediately by an unloaded shortening lasting 30 milliseconds. The remaining bound cross-bridges were mechanically detached by rapidly (1 millisecond) restretching the muscle fiber to its original length, after which tension redeveloped. The rate constant of monoexponential *k*_*tr *_was determined by fitting the rise in tension to the following equation: *F *= *F*ss(1 - e^-^*k*^tr·^*t*), where *F *is force at time *t *and *k*_tr _is the rate constant of tension redevelopment.

## Abbreviations

dHPLC: denaturing high-performance liquid chromatography; MHC: myosin heavy chain; NM: nemaline myopathy; PCR: polymerase chain reaction.

## Competing interests

The authors declare that they have no competing interests.

## Authors' contributions

MWL interpreted the clinical information, performed the pathological analysis, and prepared the manuscript. CAO and HG performed the contractile studies and composed the section of the results and discussion pertaining to contractile performance. VLL, KP, and CWP carried out the molecular genetics analyses, assisted in analyzing the clinical and genetic data and composed sections of the introduction and discussion related to these topics. KC performed genetic analysis on the patients and created the figure related to these data. AHB conceived of the study, participated in its design and data interpretation, and helped to draft the manuscript. All authors read and approved the final manuscript.
